# Transcriptomic profiling of hemp bast fibres at different developmental stages

**DOI:** 10.1038/s41598-017-05200-8

**Published:** 2017-07-10

**Authors:** Gea Guerriero, Marc Behr, Sylvain Legay, Lauralie Mangeot-Peter, Simone Zorzan, Mohammad Ghoniem, Jean-Francois Hausman

**Affiliations:** 1 Luxembourg Institute of Science and Technology (LIST), Environmental Research and Innovation (ERIN) Department, Esch/Alzette, L-4362 Luxembourg; 2 Université catholique de Louvain, Groupe de Recherche en Physiologie Végétale, Earth and Life Institute-Agronomy, Louvain-la-Neuve, B-1348 Belgium; 3 Institut National de la Recherche Agronomique, Université de Lorraine, UMR 1136, Interactions Arbres-Microorganismes, Champenoux, F-54280 France

## Abstract

Bast fibres are long extraxylary cells which mechanically support the phloem and they are divided into xylan- and gelatinous-type, depending on the composition of their secondary cell walls. The former, typical of jute/kenaf bast fibres, are characterized by the presence of xylan and a high degree of lignification, while the latter, found in tension wood, as well as flax, ramie and hemp bast fibres, have a high abundance of crystalline cellulose. During their differentiation, bast fibres undergo specific developmental stages: the cells initially elongate rapidly by intrusive growth, subsequently they cease elongation and start to thicken. The goal of the present study is to provide a transcriptomic close-up of the key events accompanying bast fibre development in textile hemp (*Cannabis sativa* L.), a fibre crop of great importance. Bast fibres have been sampled from different stem regions. The developmental stages corresponding to active elongation and cell wall thickening have been studied using RNA-Seq. The results show that the fibres sampled at each stem region are characterized by a specific transcriptomic signature and that the major changes in cell wall-related processes take place at the internode containing the snap point. The data generated also identify several interesting candidates for future functional analysis.

## Introduction

Fibre crops are important bioresources as they provide strong and long fibres (up to 100 mm in some cases^1^), also known as bast fibres. These extraxylary cells belong to the sclerenchyma, they support mechanically the phloem and are differentiated into xylan- and gelatinous-type^2^. The cell walls of xylan-type fibres are lignified, contain predominantly xylan as hemicellulose and show a typical layered structure (S1–S3) because of the different orientation of the cellulose microfibrils^2^. The gelatinous fibres, typically found in hemp bast fibres, are characterized by a thick cellulosic cell wall^1, 3^ (referred to as G-layer). Bast fibre G-layer is reminiscent of the cell walls occurring in tension wood. However, the former does not exert the same contractile function as the latter^4^. Fibre crops like textile hemp or flax (*Cannabis sativa* L. and *Linum usitatissimum* L.) are very attractive models to carry out investigations on cell wall processes, because their stems are characterized by tissues displaying remarkable differences in cell wall composition. The cortical tissues, which can be easily peeled off and separated, harbour the cellulosic bast fibres and are characterized by the occurrence of low amounts of lignin (ca. 2–7%)^1^. The core, also referred to as shivs or hurds, is instead woody. Along the stem axis it is possible to identify an empirically-determined region, called the “snap point”^5^, which marks the transition from elongation to fibre thickening (and resulting in changes in fibre mechanical properties). The fibres in the younger regions of the stem (at the top) first grow symplastically with the surrounding tissues^6, 7^, then they start to elongate actively by a mechanism known as intrusive growth, where the tip of the fibres invades the middle lamella of neighbouring cells^7–11^. This growth mechanism ensures that the number of fibres in a given transverse section of the stem increases, without changing the total number of cells. This gradient of fibre developmental stages is accompanied by a basipetal lignification gradient in the stem tissues, where genes involved in the production of phenylpropanoids, and more generally in the provision of metabolic precursors needed for lignin synthesis, are expressed at higher levels. In this respect, in hemp it was recently shown that genes involved in the non-oxidative phase of the pentose phosphate pathway and in the first reaction of the shikimate pathway were expressed at higher levels in the core tissues at the bottom of the stem^12^. The stem of fibre crops is therefore ideal to carry out high throughput molecular analyses focusing on the cell wall, because its tissue polarity and spatial lignification gradient enable the study of sequential developmental stages. Several studies have indeed been published on fibre crops, namely flax^13, 14^, jute^15–17^, ramie^18^, kenaf^19^, hemp^20, 21^ where a molecular approach was adopted to shed light on the mechanisms underlying bast fibre differentiation and development. These studies have identified important genes involved in bast fibre development, notably chitinases and cellulose synthases^13^, as well as transcription factors^15, 16^ and genes involved in secondary metabolism and monolignol-associated pathway^15, 21^. The advent of high-throughput techniques like transcriptomics has enabled huge steps forward in the study of fibre crops. For example, a very recent molecular study on flax, has shed light on the molecular mechanisms underlying advanced phases of bast fibre development, by identifying several transcription factors, as well as glycosyltransferases and unknown/not fully annotated genes^14^. Another recent study using transcriptomics/genomics has compared two varieties of jute differing in the cellulose/lignin fibre content and has demonstrated the expansion of lignin-biosynthetic genes with respect to flax^17^.

**Figure 1:**
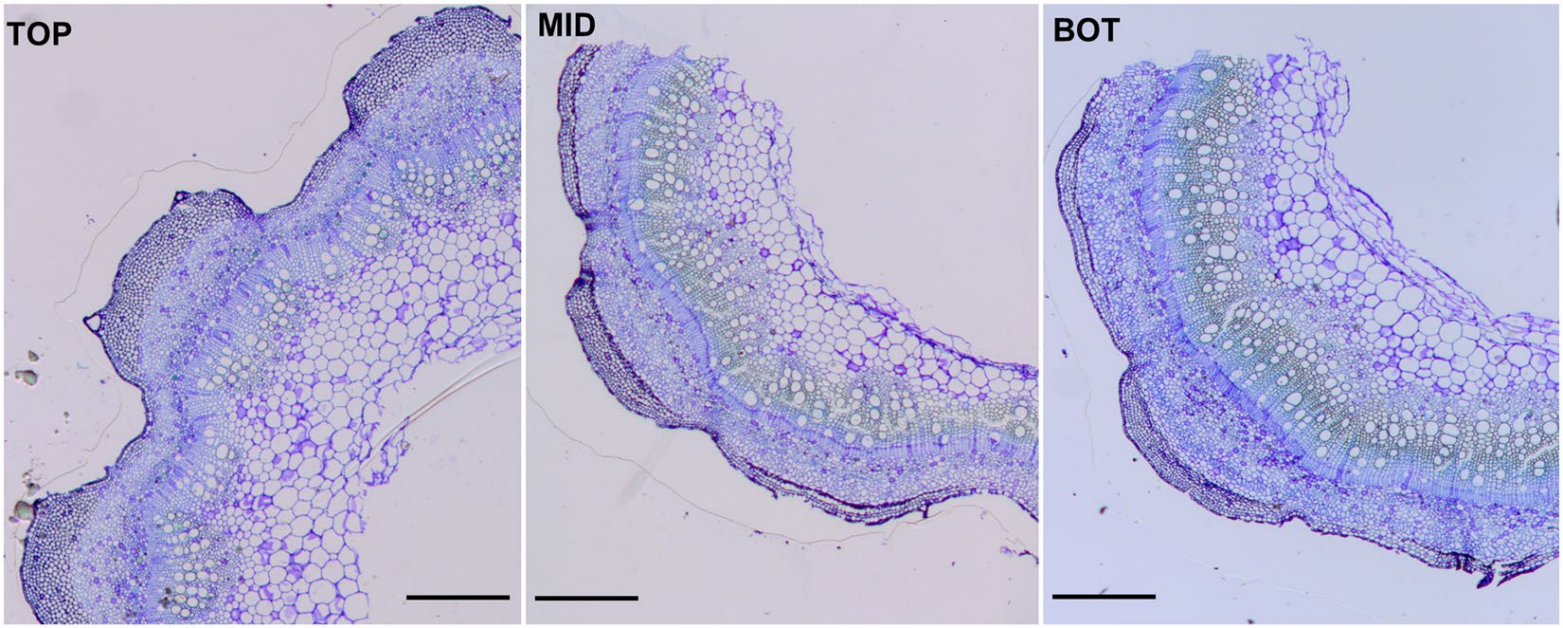
Optical microscope pictures of the different hemp stem regions (TOP, MID, BOT) analysed in this study. Staining was performed with toluidine blue. Scale bars are 500 µm.

In the light of the industrial importance that gelatinous bast fibres are receiving, we here sought to investigate, via RNA-Seq, the molecular events accompanying their development in an economically important fibre crop, textile hemp. By sampling bast fibres from the top (TOP), middle (MID) and bottom (BOT) internodes of hemp stems (Fig. 1), we show that the transcriptional signature at each stem region is unique. These results are useful to identify and characterize candidate genes involved in bast fibre elongation/thickening which can be further studied functionally and used for future biotechnological applications.

## Results and Discussion

### RNA-Seq of hemp bast fibres

To analyse the transcriptional signature of hemp bast fibres at different developmental stages, RNA-Seq was performed on fibres sampled at three stem heights, i.e. top (TOP), middle (MID, containing the snap point) and bottom (BOT). Although fibres located at the top are difficult to separate because of the lack of a well-developed tertiary cell wall (G-layer), we carefully peeled the cortical tissues from the core and got rid of the majority of epidermal/parenchymatic and xylem cells by using a mortar with pestle and ethanol 80%. We reasoned that this procedure would enable us to minimize the “contamination” from the other tissues: inspection at the microscope showed that some non-glandular trichomes, parenchyma and xylem cells were still present, however those elements were sporadic (Suppl. Fig. 1).

A *de novo* assembly of the Santhica transcriptome was here performed by merging the reads obtained from the hypocotyl (previously published transcriptome^22^) with those obtained here for the bast fibres of adult plants: we reasoned that this would enable us to better capture the cell wall-related dynamism in the isolated phloem fibres of the variety under investigation. We nevertheless validated the data by comparing the results obtained after mapping using our *de novo* assembly with those generated after mapping against the Finola transcriptome^23^ (Suppl. Dataset File). As discussed in the next paragraphs, the two mapping strategies gave comparable results.

A total of 3268 differentially expressed contigs ranging from 283 to 7095 bps was obtained after data processing (see Material and Methods); of these, 2317 are annotated (Suppl. Dataset File). The Independent Component Analysis (computed with the FastICA algorithm in MetaGeneAlyse) revealed a good separation of the different stem regions when two components were used (percentage of variance explained: 98.79%) (Suppl. Fig. 2). This indicates that the bast fibres sampled for the analysis were in different developmental stages and therefore characterized by distinct transcriptomic signatures.

The RNA-Seq data were validated using targeted RT-qPCR on a subset of 12 genes (Suppl. Dataset File): the calculated coefficient is 0.9284, which indicates a very good correlation between the RNA-Seq and RT-qPCR data (Suppl. Fig. 3).

**Figure 2:**
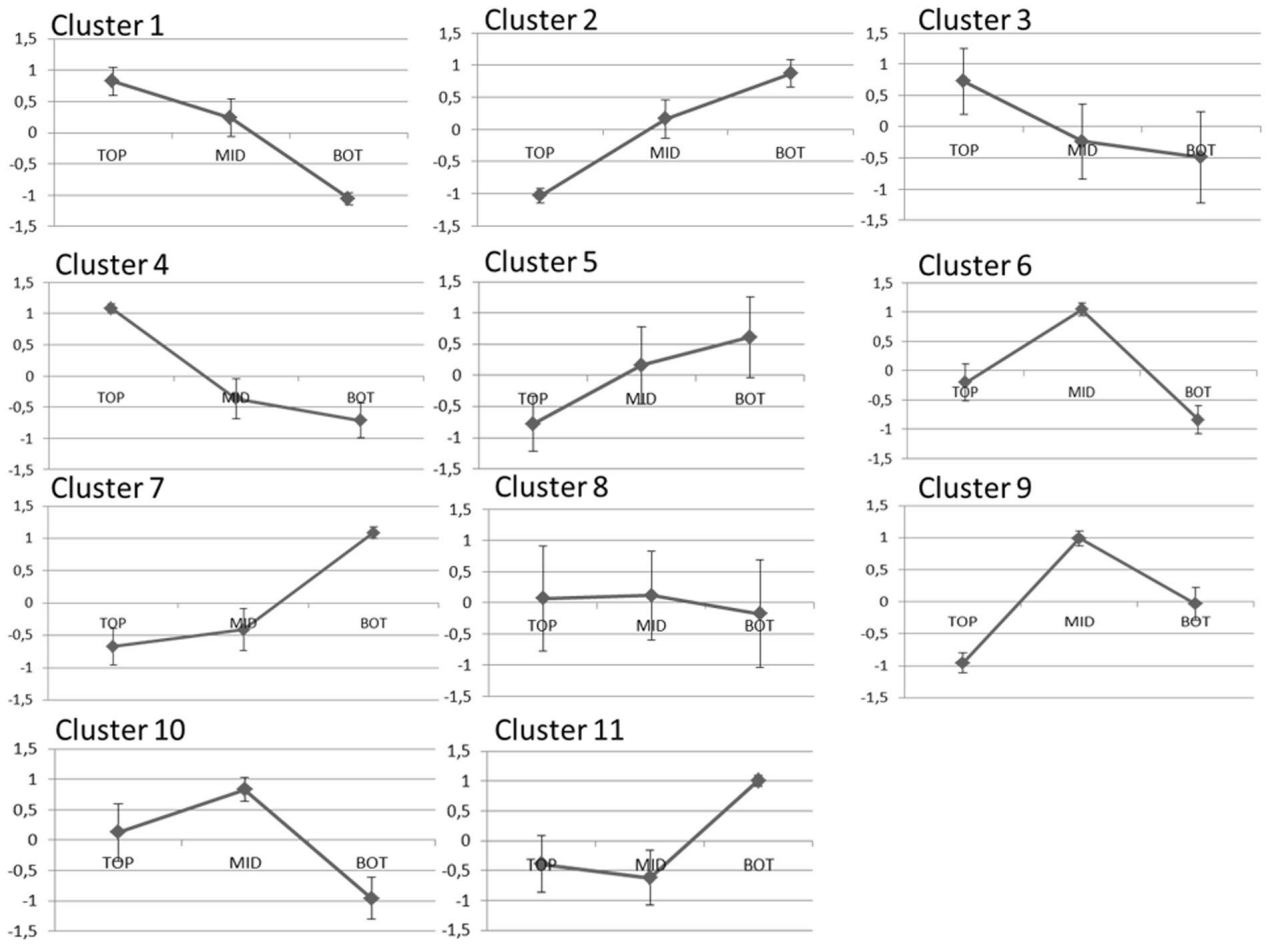
Profiles of the eleven clusters of genes obtained using a Pearson coefficient threshold of 0.75. The data represent the log_2_RPKM rescaled values± standard deviation (the rescaled values were calculated by subtracting to each contig expression value the average among the three stem regions and dividing by the standard deviation).

To get insights into the gene expression patterns, data clustering was performed using an arbitrary Pearson correlation coefficient threshold of 0.75. The analysis resulted in eleven clusters (Fig. 2); among these, cluster 1 and 2 account for the highest number of contigs (843 and 703 annotated contigs, respectively; Suppl. Dataset File). The hemp contigs can be assigned to three major expression patterns: progressive decrease in expression from the top to the bottom of the stem (clusters 1, 3, 4), progressive increase in expression along the stem axis (clusters 2, 5, 7) and maximum expression at the internode containing the snap point (clusters 6, 9, 10). Two additional trends were revealed with the clustering analysis: cluster 11 groups contigs showing a tendency towards decreased expression at the snap point, cluster 8 comprises genes showing no major changes along the stem axis.

Since the bast fibres undergo progressive cell wall thickening from the top to the bottom of the hemp stem axis, we reasoned that the bulk of cell wall-related information would be obtained by focusing on the above-mentioned three major expression patterns (clusters 1-3-4, clusters 2-5-7, clusters 6-9-10). Hereafter is a description of the key gene ontologies characterizing each stem region.

### Gene ontology enrichment analysis of the TOP region

**Figure 3:**
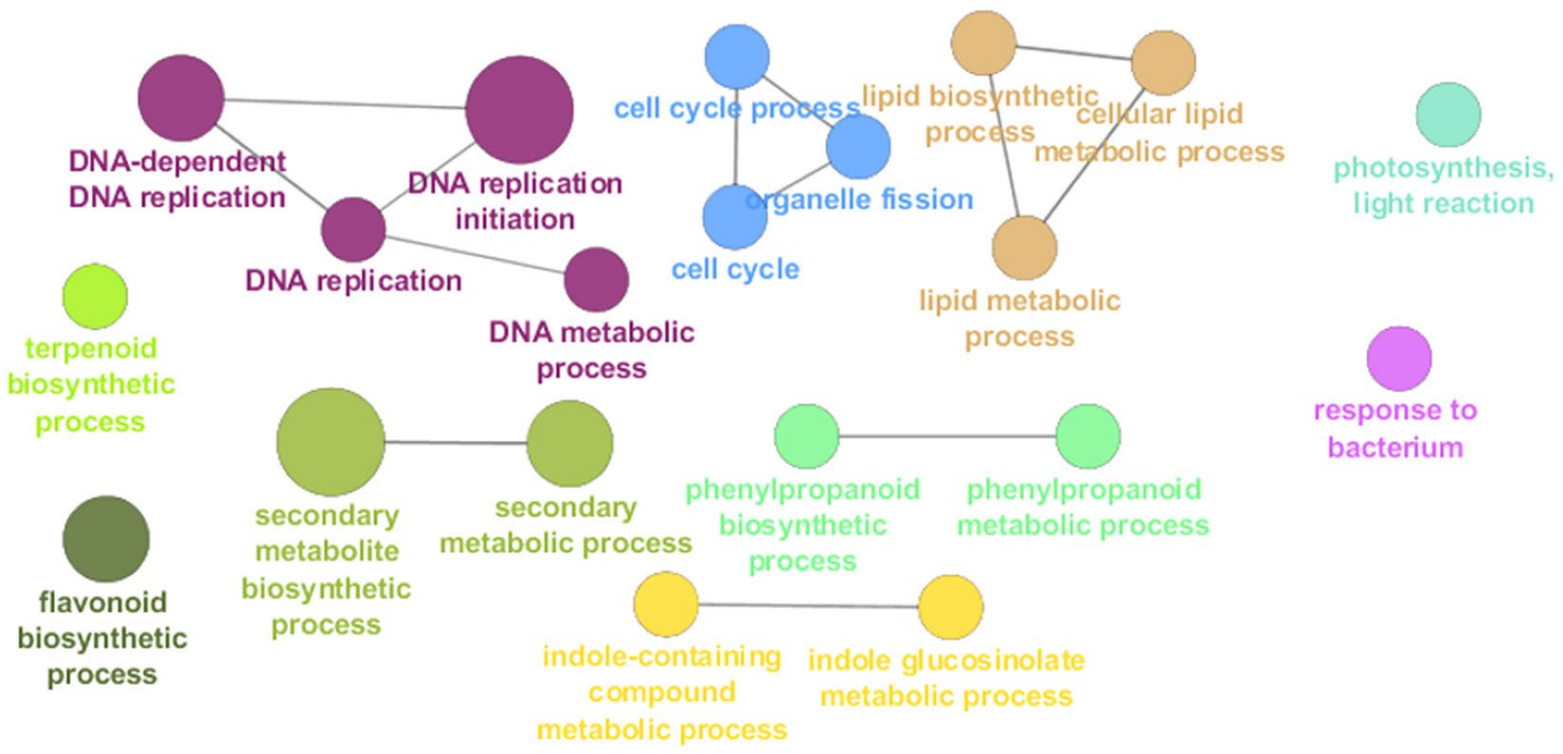
GOE analysis of the TOP region. Different colours indicate different ontologies. The bigger the circle, the higher the statistical significance.

The transcriptomic landscape of the TOP region is dominated by genes belonging to the DNA replication and cell cycle ontologies (Fig. 3), a result confirmed also by the analysis performed after mapping using the Finola transcriptome (Suppl. Dataset File). These findings are indicative of active nuclear division and are in agreement with what was previously reported in flax bast fibres^24^. Bast fibres are indeed multinucleate and during intrusive growth the number of nuclei increases, as previously documented in flax^8^. Compared to the MID and BOT respectively, *CDC6* (cell division control 6) was expressed ca. 2 and 10 times more, *DMC1* (encoding a meiotic recombination protein) was upregulated 2.4 and 7 times, *MCM2*, *4* and *5* (minichromosome maintenance protein 2, 4 and 5) were ca. 3 and 5 times more abundantly expressed, the *ORC1A* and *ORC6* genes (origin recognition complex subunit 1 and 6) were between 2.5 and 5–7 times more expressed. Additionally, the key gene *PCNA2* (proliferating cell nuclear antigen 2) involved in DNA replication^25^ was highly expressed at the TOP (ca. 99 RPKM) with respect to the BOT (18 RPKM); finally, *DRT100* (coding for a DNA-damage-repair/toleration protein) was expressed ca. 12 times more at the TOP as compared to the BOT (Suppl. Dataset File).

The enrichment of genes involved in cell division was accompanied by the over-representation of candidates partaking in the lipid biosynthetic process: several 3-ketoacyl-CoA synthase isoforms (*KCS10*, *11*, *19*) were highly expressed at the TOP, together with the β-ketoacyl reductase 1 (*KCR1*) and the fatty acid desaturases *FAD5* and *8*. The enrichment of these genes can be explained by the diffuse (intrusive) growth mechanism of bast fibres^8^: the elongation of fibres is indeed ensured by the growth of the entire surface of the cell. Notably, among the genes belonging to the lipid biosynthetic process, there are also homogentisate phytyltransferases (*HPT1*), which are involved in the biosynthesis of tocopherols. These are lipid antioxidants protecting against oxidative stress (e.g. light stress^26^).

Genes involved in photosynthetic light reaction were likewise enriched at the TOP, notably *CAB1* (chlorophyll A/B binding protein 1), *LHCB4.3* and *LHCA5* (light harvesting complex), expressed between 5 and 8 times more as compared to the BOT (Suppl. Dataset File). The TOP region was also characterized by genes involved in the response to pathogens: among them it is worth mentioning here *UVI4* (*UV-B-Insensitive 4*, encoding the protein POLYCHOME), whose overexpression was shown to induce increased resistance to a bacterial pathogen via the activation of disease resistance genes^27^. In our dataset, several pathogenesis-related (PR) genes were also upregulated at the TOP, namely *PRB1*, *PR-1-like*, *PR4* (Suppl. Dataset File).

Interestingly, the Gene Ontology term Enrichment (GOE) analysis highlighted the enrichment of genes involved in secondary metabolism biosynthetic processes too, namely terpenoids, flavonoids, indole-containing compounds and phenylpropanoids (Fig. 3). In the terpenoid biosynthetic process there are genes coding for three cytochrome P450s, i.e. *CYP76C1* and *C2* and *CYP82G1*: CYP76C1 and C2 are involved in floral linalool metabolism^28^, while CYP82G1 is responsible for the synthesis of homoterpene volatiles in *Arabidopsis* ^29^. Hemp trichomes are factories producing several terpenes contributing to the plant peculiar aroma^30^, however these metabolites can also be found in resin ducts. Since we observed the presence of non-glandular trichomes in our fibre samples, we speculate that these genes may be expressed in “contaminating” resin ducts present in our bast fibre samples.

Three genes encoding UDP glucosyltransferases were present in the “flavonoid biosynthetic process” ontology: *UGT73C1* and *C7* and *UGT78D2*. The first two belong to group D, whose members are related to stress-inducible response^31^; notably, UGT73C1 was shown to glucosylate cytokinins (*trans*-zeatin and dihydrozeatin^32^) and may therefore play a role in hormone homeostasis. UGT78D2 glucosylates instead flavonols in the C3 position and is strongly co-regulated with flavanone 3-hydroxylase (F3H, participating in the conversion of *p*-coumaroyl CoA to kaempferol/quercetin^33^), whose gene was also upregulated at the TOP in our dataset (Suppl. Dataset File).

Two genes encoding cytochrome P450s, i.e. *CYP81D1* and *D8* and two MYB transcription factors (TFs), *MYB34* and *MYB122*, were present in the indole-containing compound metabolic process: *MYB34* and *122* are two of the three TFs reported to control indole glucosinolate biosynthesis^34^. These TFs respond to phytohormones in different manners: MYB34 responds to both abscisic acid (ABA) and jasmonate (JA), while MYB122 plays a minor role in indole glucosinolate biosynthesis upon ethylene (ET) and JA signalling^34^. Given the role of indole glucosinolates in defence responses upon mechanical damage (e.g. herbivore attack^35^) and since the phytohormone JA is considered “the wound hormone”^36^, it is tempting to speculate that a mechanism involving the synthesis of JA and the subsequent activation of indole glucosinolates may be involved in the intrusive growth phase of bast fibres. The role of phytohormones in bast fibre intrusive growth remains to be confirmed.

We cannot exclude that the presence of genes belonging to the phenylpropanoid biosynthetic ontology (i.e. the laccases *LAC11* and *17*, the cinnamyl alcohol dehydrogenase *CAD9*) may be (partly) due to the traces of contaminating xylem tissues during the separation of fibre-rich peels from the TOP stem region (Suppl. Fig. 2, inset). The xylem tissue present is however in lower amount compared to the fibres; hence these transcripts may reflect an actual gene network linked to the phenylpropanoid metabolism in the TOP bast fibres. In this respect it should be noted that hemp bast fibres contain ca. 4% lignin^21^ and that a previous study highlighted the presence of transcripts associated with the secondary metabolism (namely peroxidases, methyltransferases) in the bast fibres sampled at the TOP^20^.

Among the genes grouped in the “phenylpropanoid biosynthetic process” ontology, there are five contigs annotated as *PRR1* (pinoresinol reductase 1), whose expression was upregulated at the TOP (Suppl. Dataset File). In flax, lignomics unveiled a complex monolignol metabolism associated with bast fibre hypolignification, with the accumulation of both aglycone forms and glycosides of (neo)lignans^37^; hence *PRR1* may be involved in a similar rich monolignol metabolism in hemp bast fibres. Additionally, some lignans, as dehydrodiconiferyl alcohol, are known to regulate cell division^38^; therefore PRR1 may also partake in the synthesis of specific lignans contributing to the regulation of cell division. Recently a transcriptome analysis performed on a wild-type and mutant jute (referred to as *deficient lignified phloem fibre*) identified the presence of many isoforms (more than other bast fibre-producing plants) belonging to the monolignol and shikimate-aromatic amino acid metabolism^15^. In particular, the identification of several shikimate *O*-hydroxycinnamoyl transferases demonstrated that in jute fibres there is a shunting of the phenylpropanoid metabolism from *p*-coumaroyl-CoA towards the production of monolignols, instead of flavonoids. Hemp bast fibres are of gelatinous-type and therefore the presence in the TOP bast fibres of transcripts belonging to both the flavonoid and phenylpropanoid pathways (Fig. 3) may reflect a difference with respect to the xylan-type fibres present in jute.

Among the transcripts showing the highest FC at the TOP with respect to the MID, we would like to draw the reader’s attention on 2 genes in particular, i.e. a jacalin-like lectin domain-containing protein orthologous to *AT3G16460* and a protodermal factor 1 (*PDF1*). In maize, two jacalin-like lectin domain-containing proteins were shown to be involved in cell wall-related processes^39^; in cotton, a *PDF1* gene was found to be associated with fibre initiation and early growth phases^40^. The exact role of these genes remains to be elucidated via functional studies; however their expression pattern strongly points towards an involvement during hemp bast fibre early growth stages.

### Gene ontology enrichment analysis of the MID region

**Figure 4:**
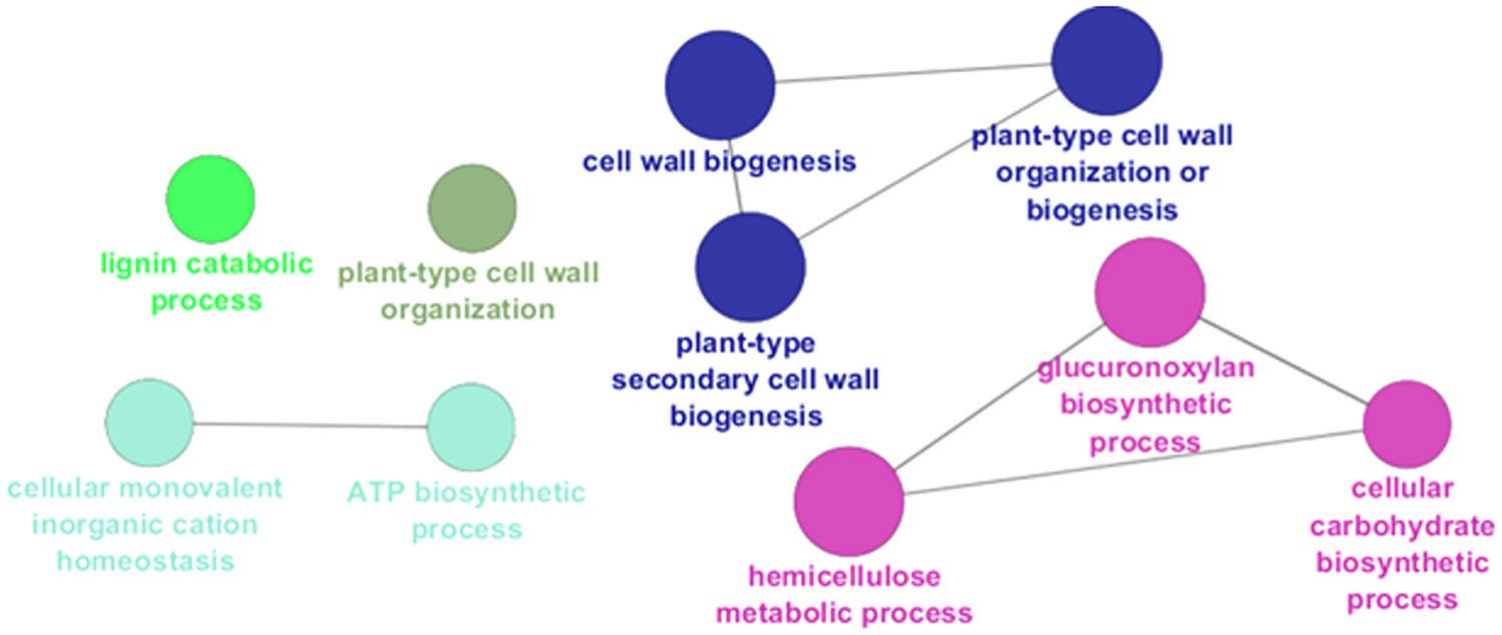
GOE analysis of the MID region. Different colours indicate different ontologies. The bigger the circle, the higher the statistical significance.

The transcriptome of the MID region is mainly characterized by processes related to secondary cell wall biogenesis (Fig. 4); this result was confirmed with the mapping against the Finola transcriptome (Suppl. Dataset File). These data are of particular relevance because they provide an overview of the key cell wall-related events responsible for the change in mechanical properties of the bast fibres observed at the snap point. In this study the MID region corresponds to the internode containing the snap point (Fig. 1); hence the fibres separated are progressively shifting from a stage of elongation to a phase of tertiary cell wall formation. According to our data, the transcriptome of the bast fibres at the MID region is characterized by genes involved in cell expansion and cell wall loosening, as well as by an active transcriptional dynamics of candidates involved in hemicellulose biosynthesis and transcriptional regulators orchestrating secondary cell wall biogenesis (Suppl. Dataset File). Alpha expansin genes (*EXPA8*, *10*, *11* and *12*) and a xyloglucan endotransglycosylase/hydrolase (*XTH33*) peaked at the MID region (*EXPA10* and *11* showed an increase in FC > 5 with respect to the TOP; Suppl. Dataset File); their higher expression is probably due to the phase of elongation characterizing the heterogeneous fibre stages at the MID. The master regulator *MYB46* and its downstream target *MYB63* were expressed 4.8 and 1.4 times more at the MID as compared to the TOP (and 3.1 and 4.3 times more at the MID as compared to the BOT): these TFs activate genes involved in xylan and lignin biosynthesis and therefore the secondary cell wall biosynthetic program^41^. Three laccases, i.e. *LAC4* (*IRX1*; contig_10035), *LAC12* (contig_15462) and 2 contigs annotated as *LAC17* (contig_19910 and contig_17371) were also highly upregulated at the MID with respect to the TOP (Suppl. Dataset File): these same genes were shown to be upregulated at older stages of development in the hemp hypocotyl^22^ and in the stem of adult hemp plants they may be associated with the peculiar lignification of the bast fibres. Bast fibres are hypolignified, however condensed guaiacyl (G) lignins were immunologically detected in the middle lamellas, cell wall junctions and S1 layer of phloem fibres in another fibre crop, flax^42^. In hemp bast fibres the presence of a similar condensed lignin was reported^43^; hence the laccases here detected may participate in the formation of condensed lignins in the cell wall of bast fibres.

In the “hemicellulose metabolic process” ontogeny there are genes involved in glucuronoxylan biosynthesis (the principal hemicellulose in dicots’ secondary cell walls^44^). *IRX8*/*GAUT12* (irregular xylem 8/galacturonosyl transferase 12)*, IRX9, IRX10*/*GUT2* (glucuronosyl transferase 2) and *PGSIP3*/*GUX2* (plant glycogenin-like starch initiation protein 3/glucuronic acid substitution of xylan 2) were upregulated 3.2, 4.5, 4.8 and 5.4 times respectively at the MID as compared to the TOP (Suppl. Dataset File). IRX8 belongs to the glycosyltransferase (GT) family 8 and is involved in the synthesis of the reducing end tetrasaccharide of glucuronoxylans, while IRX9 is a GT43 involved in xylan backbone elongation^45^; IRX10 is a GT47 also involved in the elongation of the xylan backbone^46^. GUX2 is a Golgi-localized GT8 with xylan glucuronosyltransferase activity. At the MID, the upregulation of two contigs, annotated as *ASD1*/*ARAF1* (which codes for a bifunctional α-L-arabinofuranosidase/β-D-xylosidase), was also observed (FC ca. 10 between MID and TOP). ARAF1 was shown to act *in vivo* on arabinan-containing pectins^47^ and its upregulation at the MID suggests cell wall remodelling associated with the deposition of secondary cell walls. Taking into account the upregulation of genes involved in xylan biosynthesis together with ARAF1 and considering the proof for the existence of a covalent link between hemicelluloses and pectins via an arabinogalactan protein (APAP1^48^), it is reasonable to hypothesize the presence of a similar association in the cell walls of bast fibres. ARAF1 may therefore participate in the remodelling of pectins at the onset of secondary cell wall deposition.

A contig annotated as *IRX1*/*CesA8* showed upregulation at the MID (FC MID *vs* TOP ca. 11): this result indicates that at the internode containing the snap point major molecular events related to cell wall biosynthesis take place with, notably, the upregulation of transcripts related to both cellulosic and non-cellulosic polysaccharide deposition.

Three genes coding for H^+^-ATPases (*HA3*, *5*, *6*) were upregulated at the MID: these genes may be associated with fibre elongation via the accumulation of osmotically-active compounds maintaining turgor pressure, or the acidification of the apoplast, in a manner analogous to the mechanism invoked for cotton fibre elongation^49^.

Among the contigs encoding TFs upregulated at the MID there is *IBH1* (ILI1 binding bHLH 1 protein) with the highest FC increase in expression as compared to the TOP (ca. 34; Suppl. Dataset File). IBH1 is a negative regulator of cell elongation^50^ and its higher expression at the MID may indicate a function in the shift from elongation to thickening in bast fibres. A contig coding for a PLATZ TF also peaked at the MID (MID *vs* TOP FC > 13): PLATZ TFs were proposed to act as negative regulators of cell proliferation^51^ and may contribute to the transition of bast fibres from a phase of active division to secondary growth.

Among the contigs showing the highest FC increase between MID and TOP is a putative acid phosphatase (*AT1G04040*): notably, the corresponding protein in thale cress was found associated to the cell wall fraction^52^. This phosphatase may be involved in cell wall-related processes in bast fibres during the transition from elongation to thickening and is therefore an interesting candidate for further analysis.

### Gene ontology enrichment analysis of the BOT region

**Figure 5:**
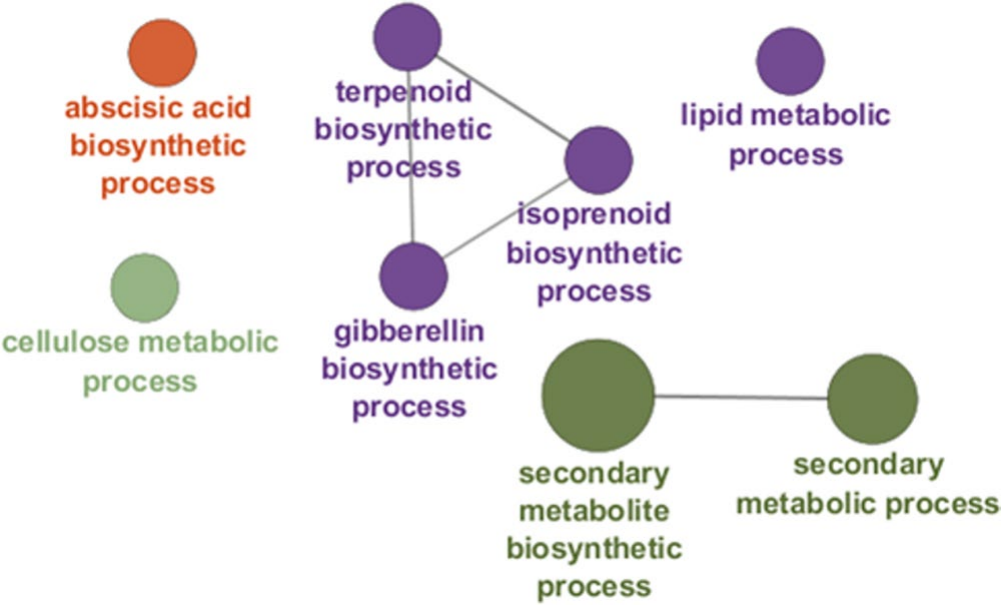
GOE analysis of the BOT region. Different colours indicate different ontologies. The bigger the circle, the higher the statistical significance.

The BOT region is characterized by ontologies related to phytohormone and non cellulosic polysaccharide biosynthesis, as well as secondary metabolic processes (Fig. 5). Mapping against the Finola transcriptome confirmed these data (Suppl. Dataset File) and additionally showed the enrichment of auxin metabolism-related genes, among which, *WAT1* (*WALLS ARE THIN1*), an auxin efflux transporter involved in secondary cell wall deposition^53, 54^.

In the “gibberellin biosynthetic process” ontogeny there are genes involved in the three steps required for gibberellin (GA) synthesis, i.e. formation of *ent*-kaurene from geranyl geranyl diphosphate, conversion of *ent*-kaurene to GA_12_ and synthesis of C_20_- and C_19_-GAs^55^. *GA1*/*CPS* (*ent*-copalyl diphosphate synthase) involved in the formation of *ent*-kaurene was expressed at the BOT > 7 times with respect to the TOP; *KAO2* (*ent*-kaurenoic acid oxidase 2), which codes for a CYP88A member catalysing the conversion of *ent*-kaurenoic acid to GA_12_^56^, showed an increase in FC > 4 as compared to the TOP; the genes encoding 2-oxoglutarate-dependent dioxygenases *GA20OX2* and *GA3OX1* showed a FC > 3 and >4 as compared to the TOP, respectively (Suppl. Dataset File). The expression *GA2OX2* also peaked at the BOT (expressed 12-times more as compared to the TOP): this gene encodes a gibberellin 2-β-dioxygenase responsible for the conversion of GA to an inactive form and participates in GA homeostasis. In addition to that, a contig annotated as a CYP714A1 (contig_19239), which participates in GA deactivation^57^, was also expressed at higher levels at the BOT with respect to the TOP (FC > 7). In the BOT region of the hemp stem, characterized by bast fibres with a thick tertiary cell wall, elongation has ceased and the homeostasis of GA levels by *GA2OX2* may represent an important regulatory mechanism. Transgenic tobacco plants in which the gene *GA2OX2* had been silenced were taller than *GA20OX1* overexpressors (the GA 20-oxidase catalyses the rate limiting step in GA biosynthesis); these results therefore show that deactivation is the key factor in the maintenance of GA homeostasis^58^. Concomitantly with the activation of *GA2OX2*, genes involved in abscisic acid (ABA) biosynthesis increased in expression at the BOT. ABA inhibits stem elongation and in our previous study it was found in higher abundance in the hemp hypocotyl at older developmental stages^22^. In our dataset, the ABA biosynthetic genes *NCED3* and *5* were both upregulated at the BOT (FC > 5 and >11 respectively, as compared to the TOP); the gene *XERICO* encoding a RING-H2 zinc finger protein and involved in ABA homeostasis^59^ was also upregulated at the BOT (FC > 4 with respect to the TOP; Suppl. Dataset File). Additionally, three contigs annotated as CYP704A2, which was proposed to be a candidate for ABA 8′-hydroxylation (i.e. catabolism^60^), were expressed at higher levels at the BOT (FC BOT *vs* TOP between 28 and 135).

A contig annotated as a CYP82C2 was upregulated at the BOT (FC difference between BOT and TOP > 16): the *A. thaliana* ortholog is involved in the modulation of tryptophan-derived secondary metabolites under conditions of high JA levels^61^.

Genes encoding putative cytochrome P450 94 members (CYP94D2 and B2) were also upregulated at the BOT (FC BOT *vs* TOP between 4.6 and 38.9): the turnover of jasmonoyl-L-isoleucine (the major bioactive form of JA) is regulated by members of this class^62^. Our results show that a molecular control over the levels GA, ABA, JA is present at the BOT of adult hemp stems.

In the “cellulose metabolic process” ontogeny it is here worth mentioning the cellulose synthase-like genes *CSLG1* and *G3* (FC > 12 and >5, respectively, as compared to the TOP); recently, in fibres of flax with tertiary cell walls, the upregulation of a *CSLG* member was observed and it was proposed that the corresponding enzyme may catalyse the synthesis of β-1,4-galactans^14^. Although the LM5 antibody recognizing β-1,4-galactans does not label hemp bast fibres^22, 63^, it may be possible that pectic galactans in hemp fibres are masked and therefore do not react with the LM5 antibody.

Genes involved in lignin biosynthesis, i.e. *PAL1* (phenylalanine ammonia lyase 1) and one contig annotated as *OMT1* (caffeic acid/5-hydroxyferulic acid *O*-methyltransferase; contig_2172) showed, respectively, a FC > 11 and >9 as compared to the TOP (Suppl. Dataset File). Likewise, one contig encoding IRX12/LAC4 (contig_13683) and a cytochrome P450 involved in lignification (CYP71A20)^64^ showed a FC increase in expression > 28 and >35 at the BOT with respect to the TOP (Suppl. Dataset File). These results are in agreement with the presence of secondary fibres in the BOT region; these fibres, notably, show a positive signal at the level of the middle lamella after phloroglucinol staining^43^. The secondary bast fibres are present, together with the primary fibres, in the cortical peels sampled at the BOT.

Several contigs coding for MYB, NAC, bHLH, bZIP and WRKY TFs were found enriched in the bast fibres at the BOT. It is important to highlight here that, in the other fibre crop *Corchorus capsularis*, a WRKY TF was upregulated during the early phases of fibre development and was suggested to be a candidate gene to improve jute fibre^16^. Additionally, in jute, bHLH, MYB-related, WRKY and NAC TFs were among the eight most abundant families^15^. These TFs may therefore have overlapping functions in the development of both xylan- and gelatinous-type bast fibres.

Among the hemp TFs, notably, there is *MYB4*, a negative regulator of lignification which may play a role in bast fibre hypolignification^37^. Among the genes encoding TFs upregulated at the BOT, it is worth mentioning a LOB-domain containing protein 4 (*LBD4*), which showed a BOT *vs* TOP increase in expression of ca. 218 (Suppl. Dataset File). LOBs are key regulators of plant organ polarity which play also a role in plant secondary growth^65^; their role in bast fibre development is to our knowledge not yet studied, however we believe that LBDs represent interesting targets in phloem fibre development. A contig annotated as *NAC047*/*SPEEDY HYPONASTIC GROWTH* (*SHYG*) peaked at the BOT (BOT *vs* TOP FC > 78): the ortholog in thale cress was shown to be associated with hyponastic leaf movements upon waterlogging via modulation of an enzyme involved in ET biosynthesis, i.e. ACC OXIDASE5^66^. ET may play a role during fibre thickening: several genes encoding ethylene-responsive TFs were indeed upregulated at the BOT, notably ERF1, WRI1, ERF106 (Suppl. Dataset File). Interestingly SHYG acts on expansin and xyloglucan endotransglycosylase/hydrolase genes to induce expansion of the petiole abaxial side. Its role in bast fibre thickening is yet to be unveiled, but it may entail a suite of cell wall-related events.

A contig encoding a RADIALIS-LIKE 6 TF was upregulated at the BOT ca. 18 fold with respect to the TOP: very recently a RADIALIS-LIKE TF was found upregulated in flax bast fibres and a role in phloem fibre development was therefore suggested^14^. RADIALIS-LIKE TFs play a role in organ (floral) symmetry; hence, together with LBDs, they may regulate aspects related with positional cues.

## Conclusions

We have here provided a transcriptional fingerprinting of bast fibres from textile hemp sampled at different stem heights, which correspond to different developmental stages. We have discussed the results using, predominantly, a cell wall angle, since the development of phloem fibres requires major modifications of the cell wall. Our study has shown that each region of the stem is characterized by distinct gene expression profiles. Young stem regions are dominated by cell cycle- and photosynthesis-related genes, together with candidates involved in the biosynthesis of specific secondary metabolites, notably indole-containing compounds and oligolignols; older internodes show enrichment of phytohormone-related genes, together with genes involved in non-cellulosic polysaccharide deposition and lignification. According to our results, the bulk of cell wall-related gene dynamics in hemp bast fibres is localized at the internode containing the snap point, where the fibres shift from a phase of elongation to thickening. The data here shown contribute to the understanding of the molecular events accompanying hemp bast fibre development and identify several genes deserving further functional study.

## Methods

### Plant material, growth conditions and optical microscopy

A hemp monoecious fibre variety (*C. sativa* cv. Santhica 27) was studied in this work. Plants were grown and sampled as previously described^12^. Briefly, after six weeks of growth in controlled chambers, samples were taken along three stem regions localized at different heights with respect to the snap point (determined empirically by gently tilting the stem apex until a kink could be observed). The top corresponds to the internode right below the apex, the middle is the internode containing the snap point and the bottom is located two internodes below the middle sample. At the time of sampling, the plants had ca. 6–7 internodes. A segment of 2.5 cm was collected in the middle of each internode to avoid too much variation in gene expression, because of the varying developmental stages of the cell types.

Fibres were separated from the shivs by peeling the cortical tissues and by quickly processing them as described previously^67^. The number of biological replicates is four with 13 plants in each replicate. Sample preparation for optical microscopy was performed as previously described^22^.

### RNA extraction and preparation of the libraries

Total RNA was extracted using a modified CTAB extraction protocol combined with an RNeasy Plant Mini Kit (Qiagen)^67^. The RNA concentration and quality were measured by using a Nanodrop ND-1000 (Thermo Scientific) and a 2100 Bioanalyzer (Agilent), respectively. All the RNAs had a RIN value between 8 and 9.

Libraries were prepared, quantified and their average size analysed as previously described^22^. The libraries were pooled at the concentration of 20 pM and sequenced on an Illumina MiSeq in five consecutive runs (MiSeq reagent kit V3, 150 cycles). Raw sequences have been deposited at the NCBI Gene Expression Omnibus (GEO) with the accession number GSE94156 (available at https://www.ncbi.nlm.nih.gov/geo/query/acc.cgi?token=itwfckosdjwdpiv&acc=GSE94156).

### Processing of the reads, mapping and RNA-Seq analysis

The raw sequences obtained were uploaded in CLC Genomics Workbench 9.0.1. Sequences were filtered as follows: sequences > 35 bps, the sequence quality score was left as default value (0.05), the maximum number of ambiguities was set to 0. Adaptor trimming was performed using the Illumina adaptor sequences, then a hard trim of 14 bps at the 5′ end and 2 bps at the 3′ end was additionally carried out, resulting in a final sequence average length of 59 bps. We had previously published a *de novo* assembly for the variety Santhica 27^22^ and proven its validity by comparing the results generated with our *de novo* assembly and with the Finola transcriptome^23^. We decided to merge the reads generated in this study with those previously obtained on the hemp hypocotyl^22^ to get a better assembly of the transcriptome of the variety under study. We therefore uploaded in CLC Genomics Workbench 9.0.1 the reads obtained previously for the hypocotyls and those obtained in the present study for the fibres from adult plants. The parameters used are: wording size was set to 20, the bubble size to 50 and minimum contig length of 300. The reads were mapped back to the assembly with a mismatch, insertion and deletion cost of 3 (stringent criteria), and a length and similarity fraction of 0.95. The assembly was then annotated using Blast2GO PRO version 3.0 against the Viridiplantae and *A. thaliana* non-redundant database. However, in Suppl. Dataset only the annotation against the *Arabidopsis* database is shown, as it was used for the subsequent Gene Ontology term Enrichment analysis (GOE) in Cytoscape (*vide infra*). For each library, the mapping was performed with a maximum hits per read of 3, a similarity and length fraction of 0.95, a mismatch, insertion and deletion cost of 3. Mapping was also performed using the transcriptome of the variety Finola^23^, as previously described^22^. The expression values were then calculated using the RPKM method^68^.

The expression values were subjected to an ANOVA statistical test with three groups (TOP, MID, BOT), each composed of four biological replicates and, subsequently, to a false discovery rate (FDR) correction. Only the genes showing a corrected *p*-value < 0.05 were retained for downstream analysis. The obtained data were further filtered by removing those genes showing a maximum value of the means < 1 RPKM (this was done with the purpose of removing those contigs showing negligible changes in gene expression) and a maximum FC > 4 in absolute value. A total of 3268 contigs was obtained (Suppl. Dataset File).

### Primer design

Primers were designed using Primer3Plus (http://www.bioinformatics.nl/cgi-bin/primer3plus/primer3plus.cgi/) and verified with the OligoAnalyzer 3.1 tool from Integrated DNA technologies (http://eu.idtdna.com/calc/analyzer). Primer efficiencies were checked via qPCR using a serial dilution of cDNA (from 10 ng to 0.0032 ng/µl). The primer sequences, amplicon length and Tm, amplification efficiencies and R^2^ are indicated in Suppl. Dataset File.

### RNA-Seq validation with RT-qPCR

The RNA extracted using the above-mentioned protocol was retrotranscribed into cDNA using the ProtoScript II RTase (NEB) and random primers, according to the manufacturer’s instructions. The cDNA was diluted to 2 ng/µl and 2 µl were used for the RT-qPCR analysis in 384-wells microplates (10 µl final volume). An automated liquid handling robot (epMotion 5073) was used to prepare the microplates. The expression of each target gene was normalized using 2 reference genes (clathrin and F-Box), after screening the 12 reference genes described previously^12^. To check the specificity of the amplicons, a melt curve analysis was performed. The expression of the genes was calculated using qBase^PLUS^ ^69^ by using the above-mentioned 2 reference genes.

### Bioinformatic analysis

The annotation of the putative transcription factors (TFs) in the *de novo* assembly was carried out with PlantTFcat^70^ (http://plantgrn.noble.org/PlantTFcat/), which gave a total of 2484 TFs (Suppl. Dataset File). The ICA was performed with the on-line program MetaGeneAlyse v1.7.1^71^ (http://metagenealyse.mpimp-golm.mpg.de/). The Gene Ontology term Enrichment analysis (GOE) was performed as previously described^72^ using Cytoscape (v3.4.0) with the ClueGO v2.3.2 plugin^73^ (*p*-value < 0.05, Benjamini-Hochberg enrichment, gene ontology from level 3 to 8, kappa score set at 0.6). RNA-Seq RPKMs were log_2_ transformed and loaded for clustering and expression profile analysis in a data analytics software developed in-house. The software includes a Web-based user interface providing interactive data visualisation in the form of a parallel coordinates plot synchronised with 2D scatter plots of PCA projections; the user interface is backed by an R server providing the necessary statistical analyses, in particular correlation clustering and PCA projection of multidimensional data. The software allowed us to configure, execute and visually analyse the RNA-Seq RPKMs; notably, with it we were able to identify the clusters of genes shown in Fig. 1.

## Electronic supplementary material

Supplementary Information

Supplementary Dataset
