# Supplementary material for: Transcriptomic profiling of hemp bast fibres at different developmental stages
Source: Sci Rep. 2017 Jul 10;7:4961. doi: 10.1038/s41598-017-05200-8 (PMC5504027; doi:10.1038/s41598-017-05200-8)
Supplement: Supplementary file 1 — Supplementary Information [file 41598_2017_5200_MOESM1_ESM.pdf]

Supplementary Information

Transcriptomic profiling of hemp bast fibres at different developmental stages

Guerriero Gea[1*], Behr Marc[1,2], Legay Sylvain[1], Mangeot-Peter Lauralie[1,3], Zorzan Simone[1], Ghoniem Mohammad[1], Hausman Jean-Francois[1]
[1] Luxembourg Institute of Science and Technology (LIST), Environmental Research and Innovation (ERIN) Department, Esch/Alzette, L-4362, Luxembourg.
[2] Université catholique de Louvain, Groupe de Recherche en Physiologie Végétale, Earth and Life Institute-Agronomy, Louvain-la-Neuve, B-1348, Belgium.
[3]Present address: Institut National de la Recherche Agronomique, Université de Lorraine, UMR 1136, Interactions Arbres-Microorganismes, Champenoux, F-54280, France.
[*]Author to whom correspondence should be addressed: gea.guerriero@list.lu

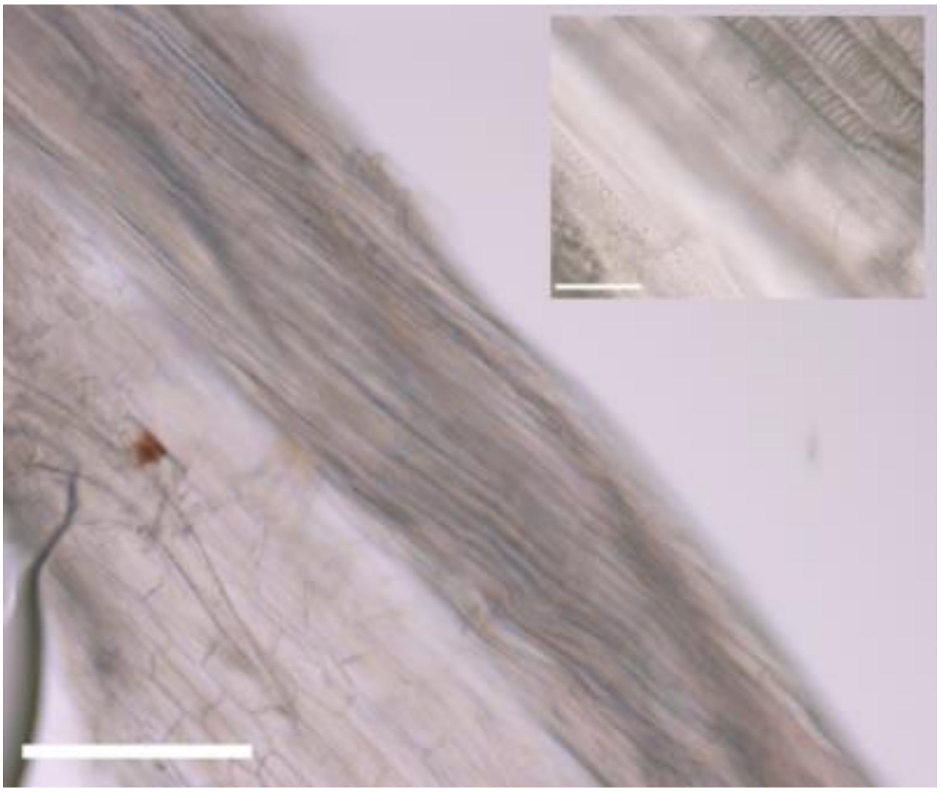

Supplementary Figure 1: Microscopic inspection of fibres isolated from the TOP region of hemp stems. In the bast fibre preparation, some sporadic non-glandular trichomes, parenchymatic and xylem cells (annular and pitted; inset) were present. Bars are 100 μm and 50 μm in the inset.

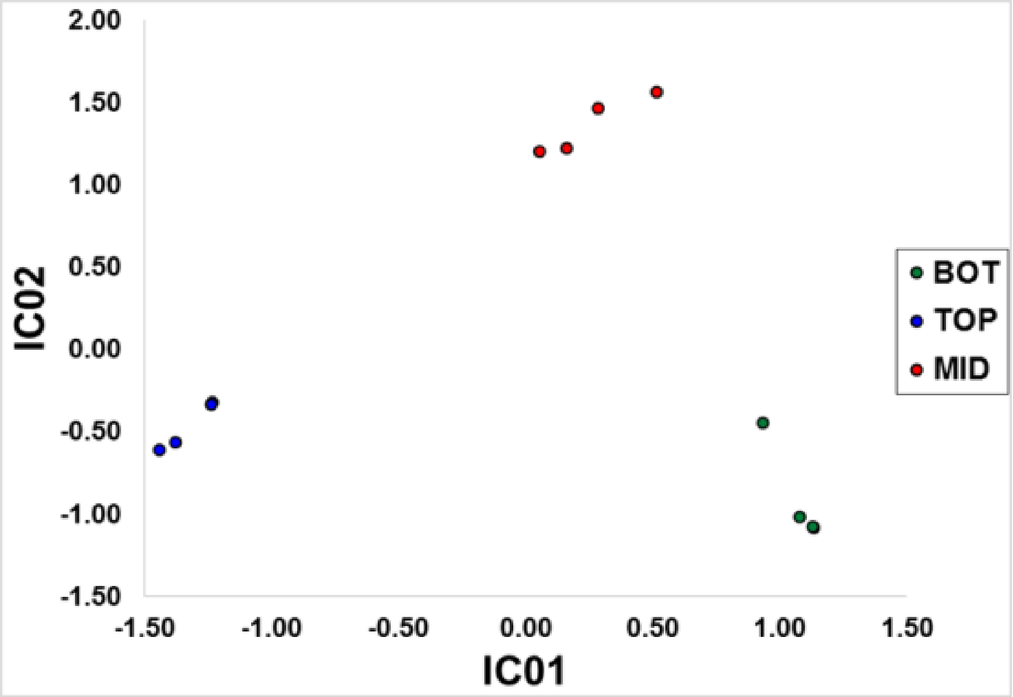

Supplementary Figure 2: ICA of the four biological replicates for the bast fibres sampled at the top (TOP, blue), middle (MID, red) and bottom (BOT, green).

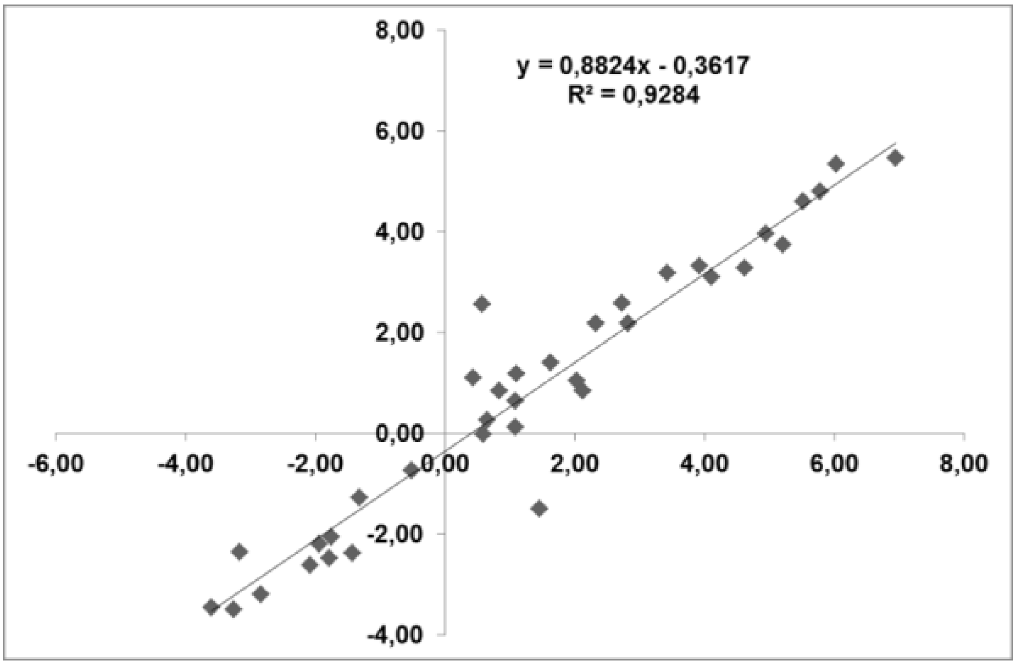

Supplementary Figure 3: Validation using RT-qPCR. The analysis was carried out on 12 representative genes (details in Supplementary Dataset File).
